# Influence of Stabilizer on the Development of Luteolin Nanosuspension for Cutaneous Delivery: An In Vitro and In Vivo Evaluation

**DOI:** 10.3390/pharmaceutics13111812

**Published:** 2021-10-30

**Authors:** Mohammed Elmowafy, Khaled Shalaby, Mohammad M. Al-Sanea, Omnia M. Hendawy, Ayman Salama, Mohamed F. Ibrahim, Mohammed M. Ghoneim

**Affiliations:** 1 Department of Pharmaceutics, College of Pharmacy, Jouf University, Sakaka 72341, Saudi Arabia; khaled_shalabym@yahoo.com; 2 Department of Pharmaceutical Chemistry, College of Pharmacy, Jouf University, Sakaka 72341, Saudi Arabia; mmalsanea@ju.edu.sa; 3 Department of Pharmacology, College of Pharmacy, Jouf University, Sakaka 72342, Saudi Arabia; omhendawy@ju.edu.sa; 4 Department of Pharmaceutics, Faculty of Pharmacy, University of Tabuk, Tabuk 71491, Saudi Arabia; ayman1grawan@gmail.com; 5 Department of Pharmaceutics and Industrial Pharmacy, Faculty of Pharmacy (Boys), Al-Azhar University, Nasr City, Cairo 11765, Egypt; mfai482@yahoo.com; 6 Department of Pharmacy Practice, College of Pharmacy, Al Maarefa University, Ad Diriyah 13713, Saudi Arabia; mghoneim@mcst.edu.sa

**Keywords:** luteolin, stabilizers, nanosuspension, skin delivery

## Abstract

Luteolin is a natural drug used as an antioxidant and anti-inflammatory, but unfortunately, it possesses low water solubility, which hinders its delivery via the skin. The main objective of this study was to prepare a luteolin-loaded nanosuspension by the antisolvent precipitation/sonication technique and study the effects of four stabilizers (two nonionic stabilizers, Pluronic F127 and Tween 80, and two polymeric stabilizers, HPMC and alginate) on the physicochemical properties of the prepared formulations. The selected formulations were incorporated into a gel base to evaluate their skin permeability and anti-inflammatory efficacy. The particle size was in the nanosize range (in the range from 468.1 ± 18.6 nm to 1024.8 ± 15.9 nm), while the zeta potential was negative and in the range from −41.7 ± 6.3 mV to −15.3 ± 1.9 mV. In particular, alginate-stabilized nanosuspensions showed the smallest particle size, the highest zeta potential value, and excellent stability due to the dual stabilizing effects (electrostatic and steric effects). The DSC results revealed a less crystalline structure of luteolin in lyophilized NS2 and NS12. Formulations stabilized by 1% Pluronic (NS2) and 2% alginate (NS12) were incorporated into a carbopol 940 gel base and showed good organoleptic character (homogenous with no evidenced phase separation or grittiness). In vitro dissolution studies showed that NS12 enhanced luteolin release rates, indicating the effect of particle size on the drug release pattern. On the other hand, NS2 showed enhanced skin permeability and anti-inflammatory effect in a carrageenan-induced paw edema model, revealing the surface activity role of the stabilizers. In conclusion, while alginate increased the nanosuspension stability by means of dual stabilizing effects, Pluronic F127 improved the skin delivery and pharmacodynamic efficacy of luteolin.

## 1. Introduction

Luteolin (3,4,5,7-tetrahydroxyflavones) is a natural flavonoid with excellent therapeutic effects. It is frequently present as glycosylated forms in several vegetables such as green pepper, chamomile flower, and honeysuckle flower [1]. Flavonoids, a class of phenolic compounds, are present in several plants, showing numerous pharmacological activities such as anti-carcinogenic, antioxidant, and anti-inflammatory activity [2]. In addition, luteolin was reported to control arthritis as it can inhibit tumor necrosis factor alpha, interleukin-1b, and suppression of activation of necrosis factor-iB. It was thus considered as a gold candidate for the treatment of arthritis. However, luteolin is classified by the Biopharmaceutical Classification System (BCS) as a Class II drug. It has low water solubility, which results in low penetration and permeation into or through the skin.

Nanosuspensions are suspensions with drug particle size ranging from dozens to hundreds of nanometers. Nanosuspensions are considered as promising systems that could improve dissolution velocity due to their large surface area and increase the solution saturation of sparingly soluble actives [3]. In addition, the lack of carrier gives nanosuspensions high drug loading, significantly higher than that of other carrier-based nanoparticles. The techniques utilized for the fabrication of nanosuspensions are mainly categorized into two method types: top-down and bottom-up techniques. The top-down techniques usually decrease the particle size without the inclusion of any organic solvent. Such techniques include milling (jet mill and ball mill) and high-pressure homogenization. Owing to the high energy input, these methods are not suitable for heat-sensitive substances. Additionally, there are restrictions to the fabrication of nanometric particles below submicron size. Besides this, crystal deformation may occur as a result of the high energy input [4,5]. On the other hand, the bottom-up methods utilize the precipitation of nanoparticles from a saturated or unsaturated drug solution. Such techniques include antisolvent precipitation, solvent evaporation, chemical precipitation, and supercritical fluid approaches. These techniques involve a low energy input compared to top-down methods [6]. Concisely, APIs are firstly dissolved in organic solvent, which is then quickly added into the antisolvent solution to precipitate the nanoparticles with the assistance of a polymer and/or surfactant [7]. Antisolvent precipitation was described as a simple and cost-effective method with scale-up potential [8]. A nanosuspension is usually developed in aqueous medium to which a stabilizer is added. Different types of stabilizer are frequently used, such as ionic stabilizers, non-ionic surfactants, and polymers. The stabilizer type and concentration are critical to the properties of the final formulation [9,10] as the stabilizer can prevent instability processes, such as particle aggregation, sedimentation, and Ostwald ripening [11]. So, the inclusion of stabilizers at the particles’ medium interface offers electrostatic repulsion or steric effects, which precludes the aggregation of particles [12].

Therefore, the aim of the present work was to explore the effects of four different stabilizers in three concentrations for the preparation of nanosuspensions; we chose two nonionic surfactants (Pluronic F127 and Tween 80) and two polymeric stabilizers (HPMC and alginate) in order to explore the influence of various stabilizers and their concentrations on the physicochemical properties of LT-loaded nanosuspensions. The selected formulations were subjected to further characterization, ex vivo permeation, and in vivo performance evaluations.

## 2. Materials and Methods

### 2.1. Materials

Luteolin (LT), Pluronic F127, and sodium alginate were purchased from Sigma Aldrich (Mumbai, India). Hydroxypropylmethyl cellulose (HPMC) and Tween 80 were purchased from Loba Chemie (Mumbai, India). All other chemicals were of analytical grade.

### 2.2. Preparation of LT-Loaded Nanosuspension Formulations

**Figure 1:**
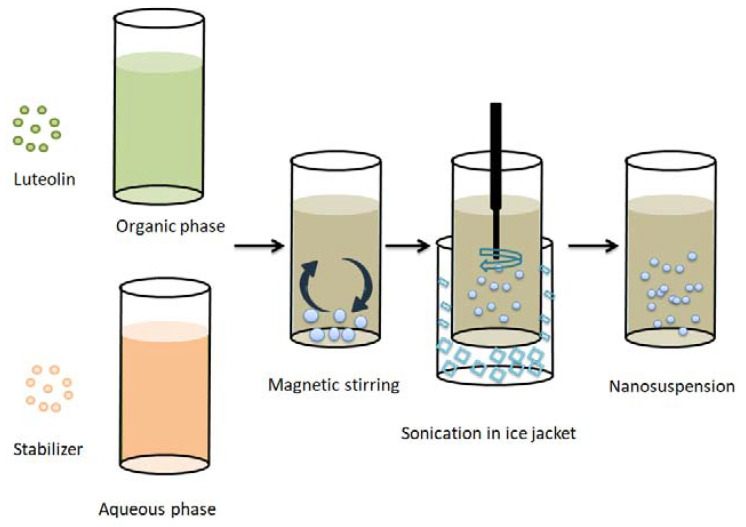
Schematic representation of the antisolvent/precipitation sonication method used for the preparation of LT-loaded nanosuspensions.

All the formulations were prepared by an antisolvent precipitation–ultrasonication technique [13] with different types of stabilizers in different concentrations (Table 1). As illustrated in Figure 1, the organic phase was prepared by dissolving 25 mg LT in ethanol (5 mL) and stirring until the drug dissolved completely. The aqueous phase was prepared by dispersing the stabilizers in 25 mL distilled water. Both the organic and aqueous phases were separately passed through 0.45 µm filters (Whatman, Kent, UK) and cooled at 4–8 °C. After that, the organic phase was rapidly added to the aqueous phase under magnetic stirring for 10 min, allowing LT particles to precipitate directly from the antisolvent. Then, primary suspensions were ultrasonically homogenized (Crest Ultrasonic Corp, Trenton, NJ, USA) to produce smaller particle size. The samples were surrounded by ice jackets to avoid temperature rise during sonication. Then, the prepared batches were stirred at ambient temperature for 24 h to evaporate the organic solvent residues.

### 2.3. Particle Size and Zeta Potential

The average particle size, polydispersity index (PDI), and zeta potential of the freshly prepared formulations were measured by a dynamic laser light scattering method using a Zetasizer (Malvern Zetasizer Nano ZS90, Worcestershire, UK) at room temperature. All measurements were performed in triplicate.

### 2.4. Short-Term Physical Stability Test

Short-term stability studies were performed to evaluate the particle size, polydispersity index (PDI), and zeta potential of the formulations when freshly prepared and after 1, 7, and 30 days at ambient temperature and protected from the light.

### 2.5. Morphology

The morphology of the selected formulations was investigated via transmission electron microscopy (TEM, JEOL JEM 1010S, JOEL Ltd., Tokyo, Japan). NS2 and NS12 were firstly diluted and shaken to obtain homogenous diluted dispersions. Then, the diluted samples were dropped onto a carbon-coated copper grid and allowed to dry. The dried films were fixed to the holder and photographed by TEM.

### 2.6. Differential Scanning Calorimetry (DSC)

Thermal analyses of pure LT, Pluronic F127, and alginate were performed. In addition, analyses of NS2 and NS12 were conducted using a differential scanning calorimeter (DSC3, Mettler Toledo, Switzerland) with a heating scan rate of 10 °C/min and temperature recorded between 30 °C and 350 °C.

### 2.7. Preparation of Nanosuspension Gels

Carbopol 940 (1%) was sprinkled into nanosuspension formulations in small portions and mixed by magnetic stirring (Magnetic stirrer, Thermolyne Corporation, Waltham, MA, USA) for 2 h or until thin dispersions were obtained. The stirring speed was then gradually decreased to allow foam break and to maintain good liquid turnover. After that, triethanolamine was added to neutralize the gel base and improve the gelation process.

### 2.8. Characterization of Nanosuspension Gels

The prepared nanosuspension gels were visually examined regarding their color, homogeneity, softness, appearance, ease of application, and phase separation. Formulations were also examined for pH (Hanna Instruments, Shanghai, China) by direct insertion of the apparatus electrode into the gel. All of the recordings were performed in triplicate.

### 2.9. In Vitro LT Release and Release Kinetics

The in vitro LT release from the selected formulations was evaluated using Franz diffusion cells (Logan DHC-6T Dry Heat Transdermal System). The donor compartment consisted of 1 g of the formulations (equivalent to 1 mg LT). The receptor compartment was filled with 12 mL of PBS/absolute ethyl alcohol mixture (pH = 5.5, 70:30). A cellulose acetate dialyzing membrane (M. wt. cut off: 12,000–14,000 Da, Livingstone, Australia) was mounted with the donor compartment above and the receptor compartment touching below. At predetermined time points (0.5, 1, 2, 3, 4, 5, and 6 h), aliquots of 0.5 mL were withdrawn for spectrophotometric assay at 340 nm (Genesys 10S UV–VIS, Thermo Scientific, Shanghai, China) with an equivalent volume of fresh medium added to the receptor compartment to maintain the sink conditions. All samples were measured in triplicate.

By plotting the mean cumulative LT released against time points, a zero-order model, first-order model, Higuchi’s model, and Hixson–Crowell model were applied to check for the best-fitting model depending on the correlation coefficient (*r*).

### 2.10. Time-Dependent Antioxidant Efficacy of the Release Medium

Samples withdrawn from the release media were subjected to an investigation of antioxidant activity via the 2,2-diphenyl-1-picrylhydrazyl (DPPH) assay method. Firstly, 6 mL of DPPH (25 µm) methanolic solution was mixed with 80 µL of release medium withdrawn at previously determined time points. Then, the mixtures were measured spectrophotometrically at 515 nm using ethanol as a blank [14]. Samples were evaluated in triplicate and the mean ± SD was recorded. The percentage antioxidant efficacy (or free radical scavenging efficacy) was calculated using the equation:

Percent antioxidant efficacy = [(Absorbance of DPPH − Absorbance of samples)/Absorbance of DPPH] × 100.

### 2.11. Skin Permeation

An LT skin permeation study was conducted on the dorsal skin of albino rats as a permeation membrane barrier using Franz diffusion cells in which the stratum corneum side was facing upwards into the donor compartment. The experiment was carried out under similar circumstances (regarding donor and receptor compartments) to those in Section 2.6 (in vitro LT release and release kinetics). One gram of the tested formulations was applied to the skin surface (epidermis side), and 0.5 mL aliquots were withdrawn from the receptor compartment at 30, 60, 120, 180, 240, 300, and 360 min time points for spectrophotometric quantification of LT. The animals (albino rats) used in this section were obtained from the Animal House Colony of the College of Pharmacy, Jouf University, and the experiment was ethically approved in accordance with the ethical procedures and policies of Jouf University (Approval code; 7/8/2021 -LCBE8/01/43).

### 2.12. Anti-Inflammatory Efficacy

Anti-inflammatory activity was evaluated in a carrageenan-induced hind paw edema model. Male Wistar rats (200–220 g) were arbitrarily allocated into 4 groups of 6 rats each. All groups were injected intraplantarly (0.1 mL/paw) with carrageenan solution (1%) [15] in the left hind paws, while the right hind paws were injected with saline solution and kept as controls. Groups 1, 2, and 3 were treated with conventional coarse LT gel, NS2 gel, and NS12 gel, respectively, 30 min before carrageenan solution injection. Group 4 was kept as a positive control. The thicknesses of the hind paws were measured using a dial caliper immediately before injection and at 1, 3, 6, 8, and 24 h after the injection. The difference in thickness between both paws was considered as the thickness of paw edema. The percentage edema inhibition (% OI) for edema induced by carrageenan was calculated using the following formula:

% OI = [(paw thickness of toxic group − paw thickness of treated group)/paw thickness of toxic group] × 100

### 2.13. Skin Compliance

Skin safety was assessed based on visual observation of erythema or any other superficial skin alterations. The dorsal surface of the rats was cleaned and hair was shaved. The formulations were applied using cotton pieces and allowed to absorb into the skin for 30 min. Then, the application areas were cleaned and rats were visually examined daily for up to seven days. The severity of skin lesions was evaluated daily based on the Draize scale [16] as follows:

0: no signs of erythema or any skin changes; 1: very minor erythema; 2: well-defined erythema; 3: marked; 4: very marked.

### 2.14. Statistical Analysis

In this study, one-way ANOVA was performed to statistically analyze the results using Tukey’s multiple comparison testing with GraphPad Prism v.5. software (San Diego, CA, USA). *p* < 0.05 was considered to indicate a significant difference.

## 3. Results and Discussion

### 3.1. Preparation of LT-Loaded Nanosuspensions

LT loaded nanosuspensions were successfully fabricated via the antisolvent precipitation–ultrasonication technique. Stabilizers are important in nanosuspension fabrication as they can prevent aggregation and agglomeration by increasing the activation energy of the process [17] and preserve the stability of the nanosuspension. For this, four different stabilizers in three concentrations were selected for the preparation of nanosuspensions—two nonionic surfactants (Pluronic F127 and Tween 80) and two polymeric stabilizers (HPMC and alginate)—in order to explore the influence of various stabilizers and their concentrations on the physicochemical properties of LT-loaded nanosuspensions. Selection of the stabilizer depends on several factors, such as the hydrophobicity and enthalpy of the candidate drug, surface energy, and specific interactions and hydrophobic moieties of the stabilizers [18]. Importantly, as LT is a hydrophobic drug (log *p* ~ 2.53) and as the stabilizer should be adsorbed onto the surfaces of the drug nanosuspension to offer a steric effect, the nominated stabilizer should have good affinity to the drug particle surfaces. Another thing is the skin tolerability of surfactants; it is well known that nonionic surfactants are more tolerable than ionic ones. For these reasons, we did not select ionic stabilizers.

### 3.2. Effect of Stabilizers on Particle Size and Zeta Potential

In this section, we discuss the effect of stabilizer type (non-ionic surfactants and polymeric stabilizers) and concentration (0.5%, 1%, and 2%) on the particle size and surface charge of the prepared formulations. Table 1 depicts the particle size, polydispersity index (PDI), and particles’ surface charge of all the prepared formulations. It is well established that the size and surface charge of a colloidal system not only influence the system stability [19] but also can affect drug delivery through the skin barrier [20]. Commonly, the aim of using a stabilizer (regardless of its nature) is to enhance the wettability of the hydrophobic surfaces of the drug particles, which, in turn, increases the activation energy and precludes particle aggregation [21]. It is clear that the particle size was in the nanosize range (in the range from 468.1 ± 18.6 nm to 1024.8 ± 15.9 nm), while the zeta potential was negative and in the range from −41.7 ± 6.3 mV to −15.3 ± 1.9 mV. It is clear that a low concentration (0.5%) of polymeric stabilizer was efficient in particle size reduction, and the particle size was 782.6 ± 14.3 nm. When increasing the concentration, Pluronic F127 was the most efficient at particle size reduction at 1% concentration (617.3 ± 25.6 nm), while at 2% concentration, particle size growth was observed (836.8 ± 13.9 nm) over polymeric stabilizers and Tween 80. Pluronic F127 (Poloxamer 407) is a copolymer composed of ethylene oxide (EO) and propylene oxide (PO) blocks arranged in an amphiphilic triblock assembly. Its hydrophilic lipophilic balance (HLB) is about 22 at 22 °C [22]. During the formation of the nanosuspension, Pluronic F127 could adsorb onto the surface of drug particles via the affinity between the hydrophobic blocks of Pluronic F127 and the hydrophobic LT [23], forming a steric repulsive layer [24]. At low concentration (0.5%), it appears that the concentration was not sufficient to cover the surfaces of LT particles completely. The ability of the surfactant to cover the crystal surface is particularly influenced by the molecular weight of the surfactant; the higher the molecular weight, the slower the surfactant diffusion [25]. It was suggested that by increasing the concentration of Pluronic F127 to 1%, the LT particles were completely covered by Pluronic F127, with greater numbers of hydrophobic blocks interacting with the LT surface due to their adsorption capacity on the particle surface [26], which is promoted by the polymer straight chain. In addition, Pluronic F127 is capable of fast diffusion to LT surfaces as it possesses outstanding dispersion characteristics and does not destroy the crystal structure of the drug particles [27]. At higher concentration (2%), the particle size of the nanosuspension increased. This shift is attributed to two reasons. Firstly, at a high concentration of Pluronic F127 above the critical micelle concentration, micelles will form, which has an important role in the thermal instability of nanosuspensions. However, the formed micelles could compete with monomers to be adsorbed at the drug surface, leading to decreased total interfacial adsorption and, hence, particle size enlargement [28]. Secondly, low monomer affinity to the drug particle surface results from the use of a high concentration of stabilizers [29].

Tween 80 is polyoxyethylene-(20)-sorbitan monooleate with an HLB value of 15. It shows a multiheaded structure: four extended polyoxyethylene chains (hydrophilic moieties), one of which is connected with a hydrocarbon chain (hydrophobic moiety), and the four polyoxyethylene chains are linked to a heterocyclic ring. Although it is a nonionic surfactant, Tween 80 exhibited less efficient particle size reduction than Pluronic F127 at all investigated concentrations. It is suggested that the multihead structure of Tween 80 might hinder efficient adsorption onto the drug particle surface. However, Mishra and coworkers found similar results when preparing hesperetin nanosuspensions using four types of stabilizers (Pluronic F68, Tween 80, Plantacare 2000, and Inutec SP1) and attributed their result to the occurrence of aggregation during the homogenization process of the Tween 80 stabilized nanosuspension [25]. In another study, Tween 80 required more milling time (35 min) than Pluronic F127 (30 min) to achieve similar particle size during the preparation of miconazole nitrate nanosuspensions [30]. It should be noted that increasing the concentration of Tween 80 did not lead to a significant particle size reduction. This result was in good accordance with findings obtained by Kobierski and coworkers during the preparation of resveratrol nanosuspensions [31].

Regarding polymeric stabilizers, the particle sizes of HPMC-stabilized nanosuspensions (801.2 ± 18.6) were insignificantly higher than those for Pluronic F127 and significantly lower than those for Tween 80 stabilized nanosuspensions at low concentration (5%). Alginate-stabilized nanosuspensions showed the lowest particle size (590.3 ± 12.8 nm, 504.5 ± 20.4 nm, and 468.1 ± 18.6 nm for 0.5%, 1%, and 2% concentrations, respectively) among all formulations. They also showed uniform particle distribution, expressed in their PDI values (0.23 ± 0.04, 0.27 ± 0.03, and 0.28 ± 0.05 for 0.5%, 1%, and 2% concentrations, respectively).

HPMC is a propylene glycol ether of methylcellulose prepared by the substitution of cellulosic polymer with methoxy and hydroxypropyl substitution at the 1, 3, or 6 positions of the repeating anhydroglucose units [32]. It was reported that the hydrophobic moiety of HPMC is capable of interaction with hydrophobic drugs (like ibuprofen) owing to their high affinity. This behavior leads to an open-chain-like adsorption pattern, rather than a compact/coiled structure, as verified by atomic force microscopy [33]. Increasing the concentration of HPMC did not significantly decrease particle size.

Alginate, a polysaccharide extracted from brown algae, is composed of 1,4-linked β-d-mannuronic acid (M) and 1,4 α-l-guluronic acid (G) residues. It was reported as an efficient particle-size-reducing agent during the formation of nanosuspensions [21]. Owing to its carrying of a negative charge, alginate can effectively stabilize a nanosuspension via a dual mechanism: polymeric steric repulsion and electrostatic repulsion. Increasing the concentration of alginate to 2% reduced the particle size to 468.1 ± 18.6 nm. This finding can possibly be attributed to the low concentration (0.5%) of alginate being inadequate to cover the surface of drug particles completely, resulting in weak stabilization. Aggregation and bridging flocculation are inclined to take place wherever a single polymer molecule may adsorb to more than one particle [34]. Guan and coworkers [35] demonstrated the high efficiency of alginate as a nanosuspension stabilizer in low concentration when compared to other commonly used stabilizers (polyvinyl alcohol, Pluronic F68, Pluronic F127, and sodium dodecyl sulfate).

The zeta potential value is a crucial factor for investigation of the stability of colloidal dispersions. The zeta potential value is the potential at the hydrodynamic shear plane. It depends on particle movement under the influence of an electrical field. The particle movement is particularly affected by both the surface charge and the concentration of the electrolyte of the stabilizers used [18]. The higher the zeta potential value, the greater the repulsion between adjacent similarly charged particles in the dispersion. The zeta potential results of all investigated formulations are outlined in Table 1. It is obvious that the particles of all formulations carried negative charge but with different values. By comparing the results, it could be suggested that the formulations stabilized by 1% alginate (−41.7 ± 6.3 mV) were the most stable among all investigated formulations. For Pluronic F127, Tween 80, and HPMC stabilized nanosuspensions, zeta potential values of around ±20 mV show complete stabilization of the nanosuspension [36]. As Pluronic F127, Tween 80, and HPMC stabilize the nanosuspension by steric stabilization, the zeta potential change upon increasing the concentration of these stabilizers was insignificant.

On the other hand, the zeta potential value significantly decreased upon increasing the concentration of alginate. This behavior may be ascribed to the fact that, if the concentrations of electrostatic stabilizer are higher than the plateau of the adsorption isotherm, a reduction in the diffuse layer takes place, resulting in a decline in zeta potential value [36].

### 3.3. Short-Term Physical Stability Test

**Figure 2:**
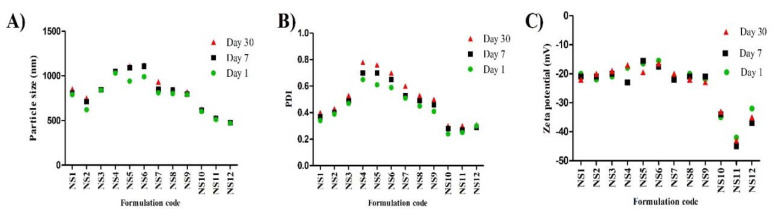
Stability studies of LT-loaded nanosuspensions over the course of 1 month (*n* = 3, ± SD): (**A**) particle size, (**B**) PDI, and (**C**) zeta potential.

**Figure 3:**
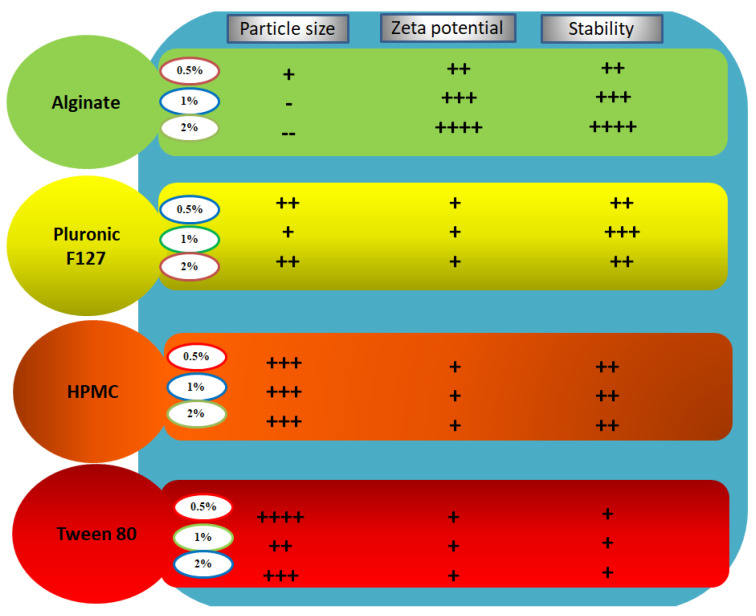
Summative representation of the results of stability studies of LT-loaded nanosuspensions.

Nanosuspensions that are temporarily stabilized or stabilized with unsuitable stabilizers will face instability-associated issues like aggregation, sedimentation, and crystalline transformation. The changes in the average particle size, PDI, and zeta potential values of all investigated formulations over the course of a month are shown in Figure 2. Based on these results, particle size growth was more pronounced in formulations NS5 and NS6 (Tween 80 stabilized formulations) after 7 and 30 days, and their particle sizes reached 1090.7 ± 32.5 and 1121 ± 29.6 nm, respectively. All other formulations looked stable, and particle size growth was insignificant. On the other hand, zeta potential values showed minimal changes during the storage period. For nanosuspensions stabilized via only electrostatic repulsion, physical stability can be obtained with a minimum zeta potential value of ±30 mV [37]. When the electrostatic repulsion mechanism is combined with the steric stabilization mechanism, a zeta potential value of nearly 20 mV is adequate to completely stabilize a nanosuspension system [18]. Generally, the aggregation phenomenon is ascribed to Ostwald ripening due to loss of the energy barrier between the particles [38]. During storage, destabilization usually takes place due to polymer bridging of the particles and surface charge neutralization. Tween 80 can stabilize the nanosuspension via steric effects. The instability of Tween 80 stabilized formulations, in comparison with the others, may be ascribed to the solubility effect of Tween 80: the higher the solubility of the drug in the stabilizer, the higher the incidence of Ostwald ripening [18]. Additionally, amorphous solid dispersion at the interface may develop when the drug is soluble in the stabilizer [39]. Pluronic F127 is a good stabilizer acting via steric stabilization. HPMC can stabilize the nanosuspension by efficient adsorption on the drug particle surface (by interaction between its hydrophobic backbone and drug hydrophobic groups), preventing aggregation [33], and by increasing the viscosity of the nanosuspension and decreasing the sedimentation rate. Viscosity-enhancing agents can also promote the development of a stereospecific blockade between the nanosuspension particles, inhibiting contact between particles. Alginate can stabilize the nanosuspension by both electrostatic and steric stabilization. Combining both mechanisms provides convenient stability to the nanosuspension, as electrostatic repulsion prevents particle size growth and the steric effect increases the particle size stability. Based on the above findings, we selected NS2 (1% Pluronic F127 stabilized formulation) and NS12 (2% alginate-stabilized formulation) for further experiments. The effect of stabilizer type and concentration on the formulation particle size, zeta potential, and stability is summarized in Figure 3.

### 3.4. Morphology

**Figure 4:**
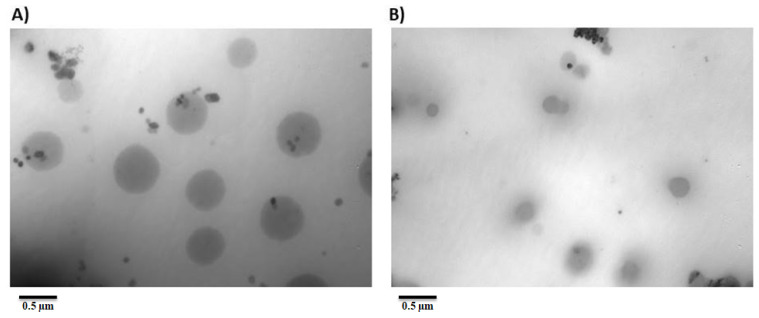
Transmission electron microscopy of (**A**) NS2 and (**B**) NS12 formulations.

The surface morphologies of NS2 (Pluronic F127 stabilized nanosuspension) and NS12 (alginate-stabilized nanosuspension) were observed by TEM (Figure 4). Both formulations presented spherical and uniform particle size, which was in accordance with Zetasizer measurements. They also showed narrow distribution. The particle sizes in nanosuspensions stabilized by alginate were significantly smaller than those stabilized by Pluronic F127, indicating the exceptional stabilizing ability of alginate. This also reflected the superiority of alginate in stabilizing the nanosuspension over Pluronic F127, as the former can stabilize a nanosuspension through both electrostatic repulsion and steric stabilization.

### 3.5. DSC

**Figure 5:**
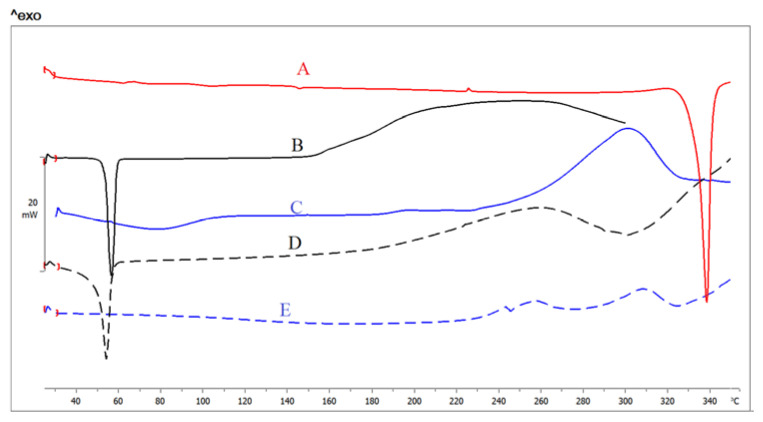
DSC thermograms of (**A**) LT, (**B**) Pluronic F127, (**C**) alginate, (**D**) NS2, and (**E**) NS12.

DSC is a key tool used to establish the crystalline/amorphous state of substances, as well as the probable interactions between drugs and carriers through finding the changes in phase transition temperature. Figure 4 shows the thermograms of LT, pure stabilizers, and NS2 and NS12. Pure LT shows a main sharp endothermic peak at 335 °C (Figure 5A), which correlates with the drug melting point [40] and crystalline state. Pluronic F127 exhibits a sharp endothermic peak at 55 °C and a broad exothermic peak at 182 °C (Figure 5B). Alginate (Figure 5C) shows a broad endothermal peak at 90 °C, representing water loss, followed by a sharp exothermic peak at 251 °C, representing degradation of polyelectrolytes via dehydration, depolymerization reactions, partial decarboxylation of the protonated carboxylic groups, and oxidation reactions of the polyelectrolytes [41]. NS2 and NS12 show a decrease in the LT endothermic peak, which was broadened and observed at lower temperature than that in the pure LT thermogram. This finding suggests a less crystalline structure of LT in lyophilized NS2 and NS12. It may be also attributed to scale-down of the particles into the nanometer range [42] as a result of the effect of efficient stabilizers. Active pharmaceutical ingredients fabricated by an antisolvent precipitation technique exhibited analogous phenomena [43].

### 3.6. Characteristics of Prepared Gels

Commonly, incorporation of a nanosuspension in a carrier gel is recommended to improve the stability for use in topical applications [44], as gel systems preclude possible aggregation of the nanosized particles. Consequently, NS2 and NS12 were incorporated into a carbopol 940 gel base. The organoleptic characteristics of the NS2 and NS12 gels were investigated through assessing the homogeneity, phase separation, and color. Both formulations showed an opaque appearance because of the greenish color of the nanosuspension. There was no evidenced phase separation or grittiness, and all the formulations looked homogenous, indicating a uniform distribution of NS2 and NS12 in the carbopol 940 gel base. This finding is essential for content uniformity, which can offer dose homogeneity. The pH values of the NS2 and NS12 gels were close to neutral and found to be in the range of 6.9 ± 0.8 to 7.2 ± 0.6. In general, these pH values are safe and anticipated to produce no cutaneous irritation [45].

### 3.7. In Vitro Release Studies

**Figure 6:**
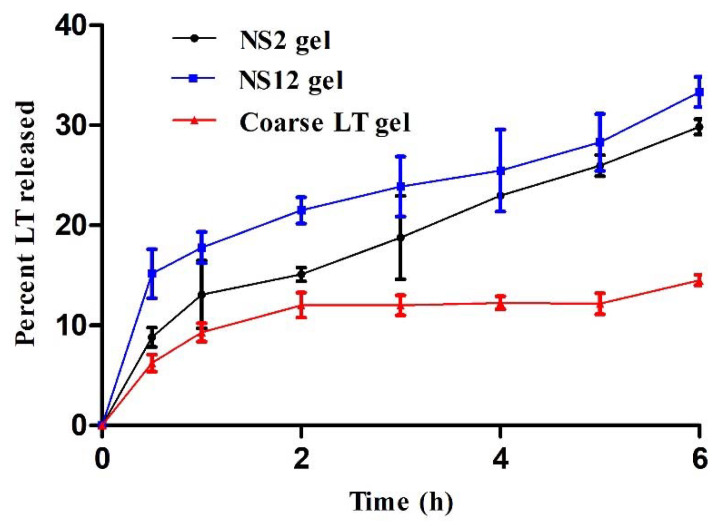
In vitro release profiles of LT from NS2, NS12, and coarse LT gels (mean values ± SD, *n* = 3).

In vitro LT release from the NS2 and NS12 gels was compared to LT release from coarse LT gel (Figure 6). All formulations were incorporated into carbopol 940. This base is considered an appropriate platform for the fabrication of various types of polymeric systems, particularly for controlled drug delivery systems [46]. As shown in Figure 6, the percentage of LT release from NS2 gel (33.3%) was higher than that from NS12 (29.8%) after 6 h. Both formulations (NS2 and NS12 gels) exhibited significantly higher LT release than the coarse LT suspension (14.5%) after 6 h. The difference in LT release might be attributed to the particle size and not to the surfactant incorporation (the particle sizes of the prepared gels showed an insignificant difference when compared to the corresponding formulations). As shown in Table 2, the particle size of the NS2 (617.3 ± 25.6 nm) formulation was higher than that of NS12 (468.1 ± 18.6 nm). However, the particle size of the coarse LT suspension was measured and found to be 2827 ± 27.9 nm. The smaller the particle size, the larger the effective surface area, which leads to a shorter diffusional layer according to the Noyes–Whitney equation [47]. According to the Ostwald–Freundlich formula, the saturation solubility of the actives increases as a function of the size of the particle [48]. On the other hand, the amorphous form of the drug is well established to have a better dissolution rate than the crystalline form. DSC studies (Section 3.5, DSC) revealed the presence of LT in amorphous form in NS2 and NS12.

Table 2 displays the kinetic data obtained by fitting the release pattern of NS2 gel, NS12 gel, and coarse LT gel through a cellulose acetate membrane to a zero-order model, first-order model, Higuchi’s model, and Hixson–Crowell model. The results revealed that the release rate of all investigated formulations was best fitted with the Higuchi diffusion model (the correlation was more linear between the percentage LT released and the square root of time), i.e., it gave the highest correlation coefficient (r) [49].

### 3.8. Time-Dependent Antioxidant Efficacy of the Release Medium

**Figure 7:**
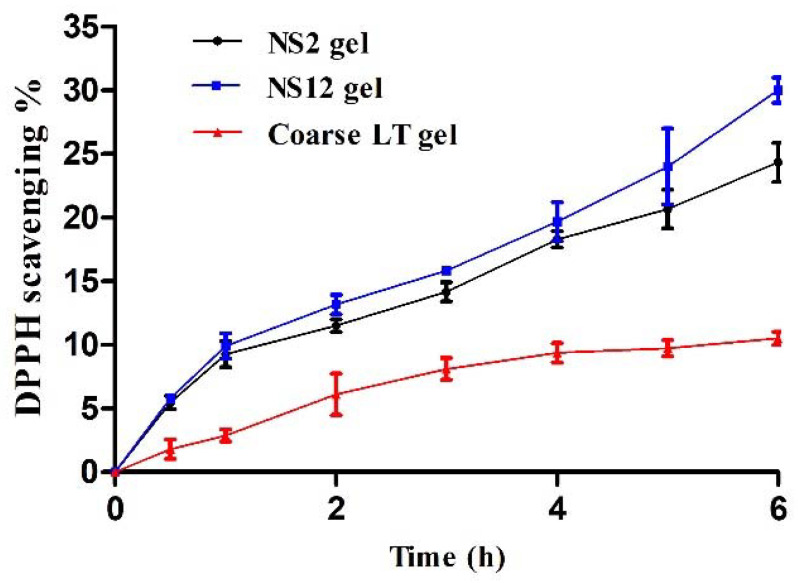
Time-dependent antioxidant activity of release media at different time intervals.

Luteolin exhibits antioxidant activity owing to the presence of ortho-dihydroxy functional groups in the B-ring and the 2,3-double bond in conjugation with the 4-oxo function of the C-ring [50]. The antioxidant efficacy associated with in vitro release media of the NS2 gel, NS12 gel, and coarse LT gel is depicted in Figure 7. Both NS2 gel and NS12 gel showed higher DPPH scavenging activity (24.3% and 30%, respectively) when compared to coarse LT gel (10.5%) after 6 h. The antioxidant activity was consistent with the concentration of LT released at the corresponding time points. This can reflect two things. The first is that LT is stable in release media, at least for the experiment period (6 h), evidenced by its retained antioxidant activity. The second is that NS gels can prolong the effect of LT via releasing effective concentrations over an extended period of time, instead of fluctuations on a short time interval.

### 3.9. Ex Vivo Skin Permeation

**Figure 8:**
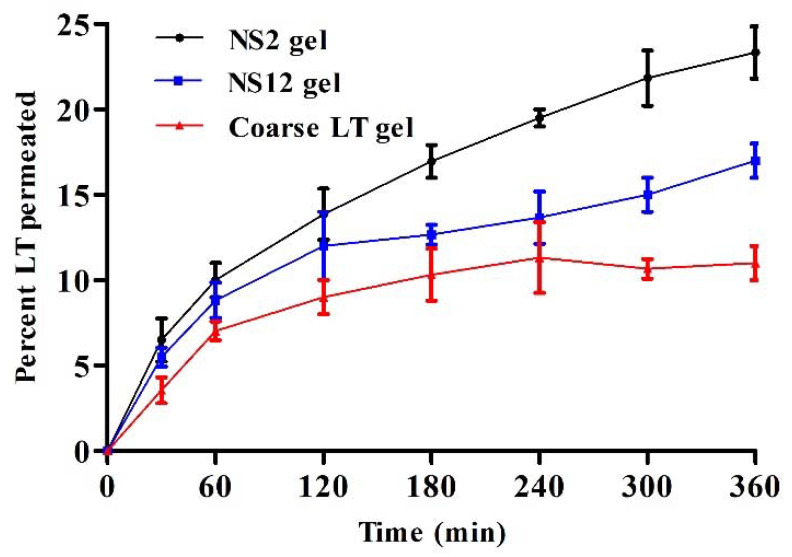
Ex vivo skin permeation of LT as a percentage through full-thickness skin samples versus time for NS2, NS12, and coarse LT gels (mean values ± SD, *n* = 3).

In the current study, a comparative representation of the average percentage of permeation was determined, and the enhancement in the permeation of LT nanosuspension gel was compared with that of coarse LT gel. The permeation profiles through full-thickness rat skin are presented in Figure 8. There was a significant difference in the percentage of LT permeated through the skin for up to 6 h after application for NS2 gel (23.3 ± 2.4%) and NS12 gel (17 ± 2.7%) when compared to coarse LT gel (11 ± 1.6%). LT is a poorly soluble drug (reported as 1.93 × 10^−5^ mol/kg at 20 °C) [40] and has a low dissolution rate. It is expected that such drugs will have low skin penetration power [20]. However, nanosizing the drug in a formulation can improve skin permeation through several mechanisms. Nanoparticulate systems possess large effective surface area, which, in turn, improves the drug saturation solubility and dissolution rate. Additionally, they create a high concentration gradient between the formulation and skin [51]. Furthermore, they can simply penetrate through the stratum corneum and permeate through the sweat glands and hair follicles to the dermal lower layers. The storage effect, which takes place with accumulation in the hair follicles, is supposed to be efficient in the penetration of nanosuspensions through the skin [52]. Although it was reported that a negative zeta potential promotes permeation into the receptor compartment, owing to skin lipid system repulsion [16], skin penetrating efficiency was more pronounced in the NS2 gel compared to the NS12 gel. This finding may be ascribed to the penetrating power of Pluronic F127. Pluronic F127 is a nonionic surface active agent and could interact with the skin, causing a disturbance in the lipid barrier in the horny layer and increasing skin permeability [53]. In addition, it was reported that particle sizes ranging from 650 to 750 nm could produce more pronounced follicular penetration than smaller or larger ones and can reach deeper skin layers [54]. The particle size of the NS2 gel was found to be in this range. The NS12 gel was an alginate-stabilized formulation. To best of our knowledge, alginate does not have any effective skin penetrating power.

### 3.10. Anti-Inflammatory Activity

**Figure 9:**
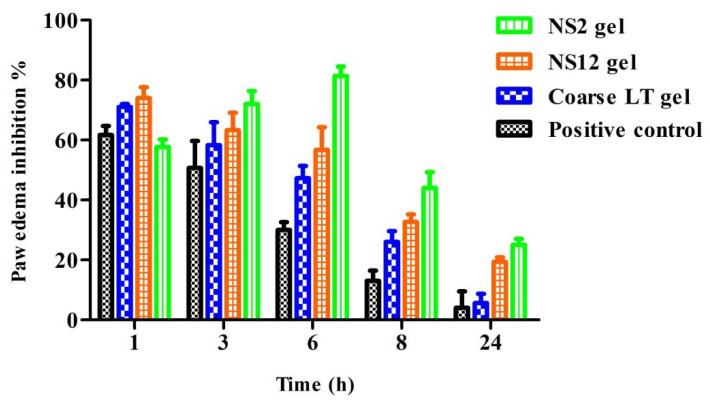
Percentages of paw edema inhibition versus time after topical application of NS2, NS12, and coarse LT gels (mean values ± SD, *n* = 6).

The anti-inflammatory effect of all investigated gels was evaluated in a carrageenan-induced rat paw edema model (Figure 9). In the positive control group, the sizes of the paw edema increased progressively and reached a peak at 8 h after carrageenan injection (percentage edema inhibition was 13.07 ± 3.4%). All investigated gels produced a decrease in paw edema volume when compared to the positive control group. The maximum edema inhibition percentage was found in the NS2 gel group at all time points, while coarse LT gel showed the lowest edema inhibition percentage. At time point 6 h, maximum edema inhibition was observed in the NS2-gel-treated group (81.33 ± 5.6%). LT can induce an anti-inflammatory effect through inhibition of nitric oxide (NO) and other inflammatory cytokines, such as tumor necrosis factor alpha (TNF-α) and interleukin-6 (IL-6), and inhibition of protein tyrosine phosphorylation and nuclear transcription factor KB (NF-KB)-mediated gene expression [55].

These results suggest that the anti-inflammatory activity of LT is improved by the nanosuspension formulation. The results were also consistent with the ex vivo permeation studies, which showed the superiority of the NS2 gel over all other investigated formulations.

### 3.11. Safety

The safety/toxicity aspects of the investigated formulations were evaluated by skin irritation test. Irritation was visually assessed after topical application of NS2, NS12, and coarse LT gel formulations. All investigated formulations produced no erythmal effects, and no irritation/redness was observed in any group. The irritation score (primary skin irritation index) was zero for all groups, which indicated their safety.

## 4. Conclusions

In the present work, different types and concentrations of stabilizers were utilized to fabricate LT nanosuspensions to satisfy the requirement for effective cutaneous delivery. It was found that the stabilizer type had a significant effect on the particle size, PDI, zeta potential, and stability. In particular, alginate stabilizer can produce a more satisfactory nanosuspension formulation in terms of particle size, PDI, zeta potential, and stability when compared to the other investigated stabilizers. This may be attributed to the dual stabilizing effect of alginate. After incorporation into a carbopol 940 gel base, the 2% alginate-stabilized nanosuspension exhibited a higher release pattern when compared to the 1% Pluronic F127 stabilized nanosuspension. On the other hand, the surfactant property of Pluronic F127 significantly improved skin permeation and anti-inflammatory activity in carrageenan-induced paw edema. Therefore, a combination of the two surfactants in an optimized ratio is suggested to develop a promising system to increase both the shelf stability and cutaneous delivery of LT. Hence, further work on such a combination is required in future studies.

## Figures and Tables

**Table 1:** Compositions, mean particle sizes, PDIs, and zeta potential values (means ± SD, *n* = 3) of different nanosuspension batches.

Code	Antisolvent	Concentration (%)	Particle Size (nm)	PDI	Zeta Potential (mV)
NS 1	Pluronic F127	0.5	782.6 ± 14.3	0.34 ± 0.04	−19.6 ± 2.9
NS 2	Pluronic F127	1	617.3 ± 25.6	0.38 ± 0.05	−22.6 ± 3.9
NS 3	Pluronic F127	2	836.8 ± 13.9	0.46 ± 0.07	−20.8 ± 4.3
NS 4	Tween 80	0.5	1024.8 ± 15.9	0.62 ± 0.02	−17.9 ± 2.8
NS 5	Tween 80	1	932.7 ± 22.9	0.59 ± 0.06	−16.2 ± 6.5
NS 6	Tween 80	2	987.2 ± 31.5	0.57 ± 0.05	−15.3 ± 1.9
NS 7	HPMC	0.5	801.2 ± 18.6	0.49 ± 0.03	−20.9 ± 2.8
NS 8	HPMC	1	793.7 ± 23.5	0.42 ± 0.05	−19.7 ± 5.7
NS 9	HPMC	2	783.9 ± 17.6	0.47 ± 0.07	−21.2 ± 7.1
NS 10	Alginate	0.5	590.3 ± 12.8	0.23 ± 0.04	−34.9 ± 12.6
NS 11	Alginate	1	504.5 ± 20.4	0.27 ± 0.03	−41.7 ± 6.3
NS 12	Alginate	2	468.1 ± 18.6	0.28 ± 0.05	−30.9 ± 8.5

**Table 2:** Kinetic parameters for the in vitro release of LT from NS2, NS12, and coarse LT gels.

Code	Zero-Order Kinetics			First-Order Kinetics			Higuchi Model			Hixson–Crowell		
	r	t_1/2_	K	r	t_1/2_	K	r	t_1/2_	K	r	t_1/2_	K
NS2 gel	0.964	11.6	4.27	0.447	2.17	0.318	0.993	18.65	11.57	0.972	12.76	0.007
NS12 gel	0.895	12	4.14	0.45	2.17	0.32	0.973	17.8	11.8	0.91	12.89	0.07
Coarse LT gel	0.817	28.5	1.74	0.483	1.97	0.350	0.938	90.1	5.2	0.824	33.3	0.02

## Data Availability

Not applicable.
